# The Analysis of Trajectory Control of Non-holonomic Mobile Robots Based on Internet of Things Target Image Enhancement Technology and Backpropagation Neural Network

**DOI:** 10.3389/fnbot.2021.634340

**Published:** 2021-03-22

**Authors:** Lanfei Zhao, Ganlin Wang, Xiaosong Fan, Yufei Li

**Affiliations:** ^1^The Higher Educational Key Laboratory for Measuring and Control Technology and Instrumentations of Heilongjiang Province, Harbin University of Science and Technology, Harbin, China; ^2^Huabei Oil Communication Co., Ltd., Cangzhou, China; ^3^Medical Cosmetic Center, Department of Dermatology, Tongji Hospital, Tongji University School of Medicine, Shanghai, China; ^4^Department of Plastic Surgery, Huashan Hospital, Fudan University, Shanghai, China

**Keywords:** backpropagation neural network, Internet of Things, image enhancement, non-holonomic mobile robot, trajectory tracking and control

## Abstract

The trajectory tracking and control of incomplete mobile robots are explored to improve the accuracy of the trajectory tracking of the robot controller. First, the mathematical kinematics model of the non-holonomic mobile robot is studied. Then, the improved Backpropagation Neural Network (BPNN) is applied to the robot controller. On this basis, a mobile robot trajectory tracking controller combining the fuzzy algorithm and the neural network is designed to control the linear velocity and angular velocity of the mobile robot. Finally, the robot target image can be analyzed effectively based on the Internet of Things (IoT) image enhancement technology. In the MATLAB environment, the performances of traditional BPNN and improved BPNN in mobile robots' trajectory tracking are compared. The tracking accuracy before and after the improvement shows no apparent differences; however, the training speed of improved BPNN is significantly accelerated. The fuzzy-BPNN controller presents significant improvements in tracking speed and tracking accuracy compared with the improved BPNN. The trajectory tracking controller of the mobile robot is designed and improved based on the fuzzy BPNN. The designed controller combining the fuzzy algorithm and the improved BPNN can provide higher accuracy and tracking efficiency for the trajectory tracking and control of the non-holonomic mobile robots.

## Introduction

As human society enters the era of science and technology, computers, and artificial intelligence have developed rapidly; machines to replace human labor to improve production efficiency have become a reality (Ma et al., 2020). However, Lv et al. (2019) also proposed that the existing network structure migrated computing tasks to the cloud, while the increase in cloud data transmission put huge pressure on the core network and affected the quality of service (Lv and Xiu, 2019). Internet of Things (IoT) is a famous object vision. Information-sensing equipment, such as sensors and electronic tags installed on the object, transmits information collected back and forth through the internet connection according to the agreed protocol. Connecting a simple robot to the internet will become valuable because it can obtain updated information about its environment from sensors or understand the user's whereabouts and the status of nearby devices (Marques et al., 2019). In short, robots integrated with IoT can use IoT data to help machines interact with each other and take necessary actions, enabling robots to communicate effectively and make appropriate decisions by themselves. The core of “IoT+Robot” is the ubiquitous sensors, cameras, and actuators embedded in the environment, as well as autonomous robots that collect data in real-time (Rehman et al., 2019). Sensors provide not only raw data but also interpretation and abstraction to some degree, which can be utilized for decision-making or high-level automation. Lv et al. (2019) applied the sensor technology of the ZigBee wireless network organization, which could provide people with a smarter and more comfortable living environment (Lv et al., 2019). Connecting machine vision systems to IoT can create powerful network functions that can recognize objects from cameras. Such functions can enhance the local nodes' intelligence and autonomy, reducing the processing load on the central server and achieving a better-distributed control architecture.

The IoT-based multi-robot collaborative operation utilizes the intelligent perception inside and outside the robot, makes timely judgments and control decisions according to the signals collected by the network, and timely issues control instructions to the robot to ensure that multiple robots complete tasks safely and efficiently (Özdemir, 2019; Michie et al., 2020). Mobile robots are highly intelligent systems that can continuously obtain information of the surrounding environment and themselves through sensors in real-time, make decisions, analyze, plan for different environments, and control the drive motors to move autonomously toward the targets, thereby completing specific tasks.

Tracking control in mobile robots' motion control has always been a sophisticated problem, which has received extensive attention from researchers in this field. Yang and Pan proposed a sliding mode control method for wheeled mobile robots, established a motion control model for mobile robots, and designed a sliding mode trajectory tracking controller, which effectively reduced the jitter of sliding mode control's input, accelerated the convergence speed, and improved the tracking accuracy (Yang and Pan, 2018). Tinh and Linh improved the online weight adjustment algorithm based on backpropagation and proposed an adaptive tracking controller based on a three-layer neural network, which could ensure the stability of the entire closed-loop system and realize the desired mobile robots' trajectory tracking performance (Tinh and Linh, 2018).

In practical applications, trajectory tracking of mobile robots should ensure versatility while ensuring stability and robustness optimization. Therefore, the focus is on the trajectory control of mobile robots. Here, a trajectory tracking method combining improved Backpropagation Neural Network (BPNN) and fuzzy neural network is proposed, denoted as fuzzy-BPNN. Then this method's feasibility and accuracy in robots' trajectory tracking and control are verified through simulation. On this basis, the IoT multi-sensor data fusion and target infrared image enhancement technology are researched. The multi-robot system can fuse the sensor data, enhance the target images, and provide new ideas for robot obstacle avoidance through fuzzy control.

## Materials and Methods

### Mathematical Kinematics Models of Non-holonomic Mobile Robots

In mechanics, mathematical equations containing coordinate parameters can express constraints. The motion constraints that mobile robots are subjected to include holonomic constraints and holonomic constraints. Holonomic constraints are restrictions on the configuration space, and non-holonomic constraints are restrictions on system motion (Chu et al., 2018). The holonomic constraint reduces the dimensionality of the configuration space. The system can transform the holonomic constraints it receives into the constraints on the position through integration during the motion. The non-holonomic constraints reduce the dimensionality of the velocity. The equations of holonomic constraints and non-holonomic constraints can be expressed as Equations (1) and (2):

(1) $$\begin{array}{l} h\left(q,t\right)=0 \end{array}$$

(2) $$\begin{array}{l} h\left(q,\overset{.}{q},t\right)=0 \end{array}$$

In Equations (1) and (2), *q* represents the coordinate vector of the system, $\overset{.}{q}$ represents the velocity vector of the system, and *t* is the time parameter.

For practical problems, the motion constraint can be transformed into a linear relationship with the system velocity $\overset{.}{q}$; that is, the Pfaffian constraint:

(3) $$\begin{array}{l} h\left(q,\overset{.}{q},t\right)=\overset{n}{\underset{i=1}{\sum }}A_{i}\left(q\right)\cdot \left(\overset{.}{q}\right)=A\left(q\right)\cdot \left(\overset{.}{q}\right)=0 \end{array}$$

In Equation (3), *A* (*q*) ∈ *R*^*m* × *n*^ represents a set of *m* velocity constraints, and $A_{i}\left(q\right)\in R^{1\times n}$ is the row vector of *A* (*q*), which is a constraint on the direction of the generalized velocity *q* of the system.

**Figure 1:**
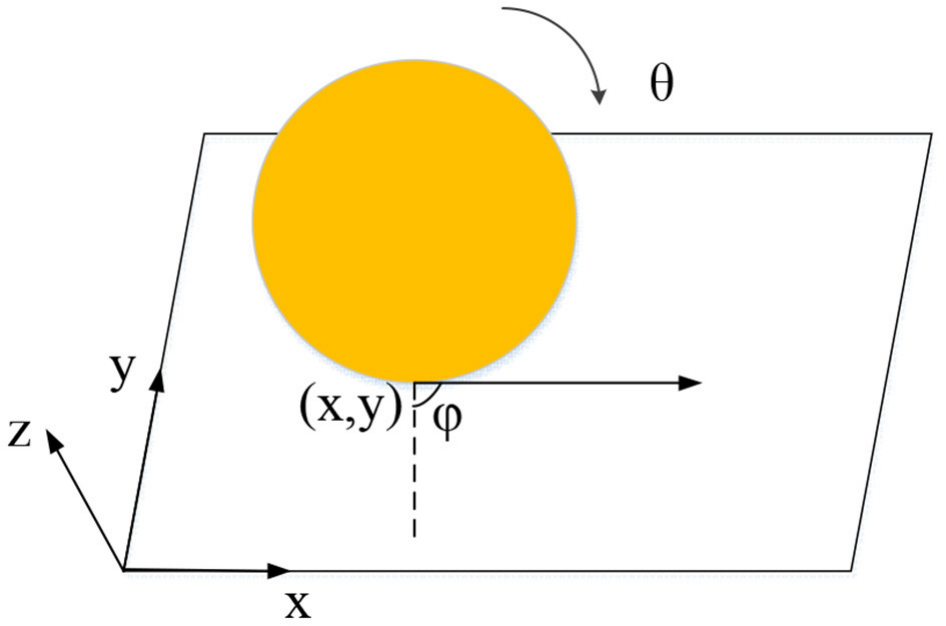
Non-slip rolling constraints of wheels on the ground.

A wheel moves on the ground, as shown in Figure 1. Four parameters can describe the wheel's configuration: the contact points *x* and *y* with the ground, the current rotation angle θ, and the forward direction Φ. If the wheel makes a non-slip motion, the direction of the wheel will always be (*cosϕ, sinϕ*). The non-slip constraint does not reduce the dimension of the wheel configuration space; that is, the wheel can reach any position on the plane. However, this constraint reduces the dimensionality of the wheel's velocity space so that the wheel can only move in direction (*cosϕ, sinϕ*) at a particular time.

Assuming that the wheel's radius is *r*, the distance between the wheel's center (*x*_*c*_, *y*_*c*_) and the ground track is always the same during the non-slip rolling motion of the linear track. The non-slip rolling between it constrains the wheel's motion and the ground, expressed as the instantaneous velocity *v* of the wheel's contact point and the ground track is zero. Therefore, the wheel's motion can be regarded as a rapid rotation around the contact point, and the constraint equation can be expressed as:

(4) $$\begin{array}{l} y_{c}=r \end{array}$$

(5) $$\begin{array}{l} A\left(q\right)\cdot \left(\overset{.}{q}\right)=\left[1\text{0 -}r\right]\left[\begin{array}{c} \overset{.}{x_{c}}\\ \overset{.}{\begin{array}{c} y_{c}\\ \overset{.}{\phi } \end{array}} \end{array}\right]=\overset{.}{x_{c}}-r\overset{.}{\phi }=v=0 \end{array}$$

Equation (4) is a geometric constraint on the wheel, and Equation (5) represents a linear motion constraint, which can be further expressed by integral:

(6) $$\begin{array}{l} x_{c}-r\phi =C \end{array}$$

In Equation (6), *C* is the integral constant.

The non-holonomic constraint equation does not have a corresponding geometric constraint. Hence, it puts no restriction on the position vector but only restrictions on the particle's velocity at each position. Therefore, the non-holonomic constraint does not reduce the number of independent generalized coordinates; instead, it only reduces the number of independent generalized velocities (Gutiérrez-Giles et al., 2018).

**Figure 2:**
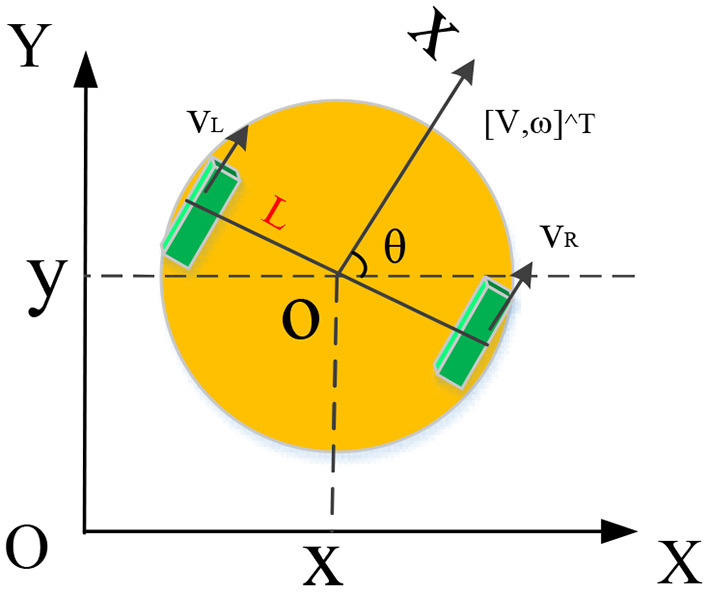
Structure of a non-holonomic mobile robot.

A non-holonomic mobile robot is also a non-holonomic system, and the non-holonomic constraint equation can reflect its motion characteristics. While the robots are moving, two physical phenomena, tire rolling and sliding, will occur when the wheels contact the ground. The structure of a non-holonomic mobile robot is shown in Figure 2. One coordinate system is the global coordinate system *XOY*; the other coordinate system is the local coordinate system *xoy* (the mass center of the robot is the origin). In Figure 2, *R* is the radius of the robot's driving wheel, θ is the robot's forward direction angle, *q* is the robot's pose, [*v ω*]^*T*^ is the robot's control quantity (linear velocity and angular velocity), *v*_*L*_ and *v*_*R*_ are the linear velocity of the robot's left and right wheels, and *L* is the distance between the centers of the robot's two driving wheels.

For error reduction, assuming that the mobile robot moves along a linear track gradually, the wheels do not slide left and right. Then the non-holonomic constraints and the kinematics model can be expressed as in Equations (7) and (8):

(7) $$\begin{array}{l} \overset{.}{y}\cos\phi -\overset{.}{x}\sin\theta =0 \end{array}$$

(8) $$\begin{array}{l} \left[\begin{array}{c} \overset{.}{x}\\ \overset{.}{\begin{array}{c} y\\ \overset{.}{\theta } \end{array}} \end{array}\right]=\left[\begin{array}{cc} \cos\theta & 0\\ \sin\theta & 0\\ 0 & 1 \end{array}\right]\left[\begin{array}{c} v\\ \omega \end{array}\right] \end{array}$$

The non-holonomic mobile robot's left and right wheels' linear velocities *v*_*L*_ and *v*_*R*_ share the following relationship with its linear velocity *v* and angular velocity ω :

(9) $$\begin{array}{l} \left[\begin{array}{c} v_{L}\\ v_{R} \end{array}\right]=\left[\begin{array}{cc} 1/R & L/2R\\ 1/R & L/2R \end{array}\right]\left[\begin{array}{c} v\\ \omega \end{array}\right] \end{array}$$

In summary, the kinematics model of the discussed non-holonomic mobile robot is:

(10) $$\begin{array}{l} \left[\begin{array}{c} \overset{.}{x}\\ \overset{.}{\begin{array}{c} y\\ \overset{.}{\phi } \end{array}} \end{array}\right]=\left[\begin{array}{cc} \frac{R}{2}\cos\theta & \frac{R}{2}\cos\theta \\ \frac{R}{2}\sin\theta & \frac{R}{2}\sin\theta \\ \frac{R}{L} & -\frac{R}{L} \end{array}\right]\left[\begin{array}{c} v_{L}\\ v_{R} \end{array}\right] \end{array}$$

### Robot Trajectory Tracking Based on Improved BPNN

**Figure 3:**
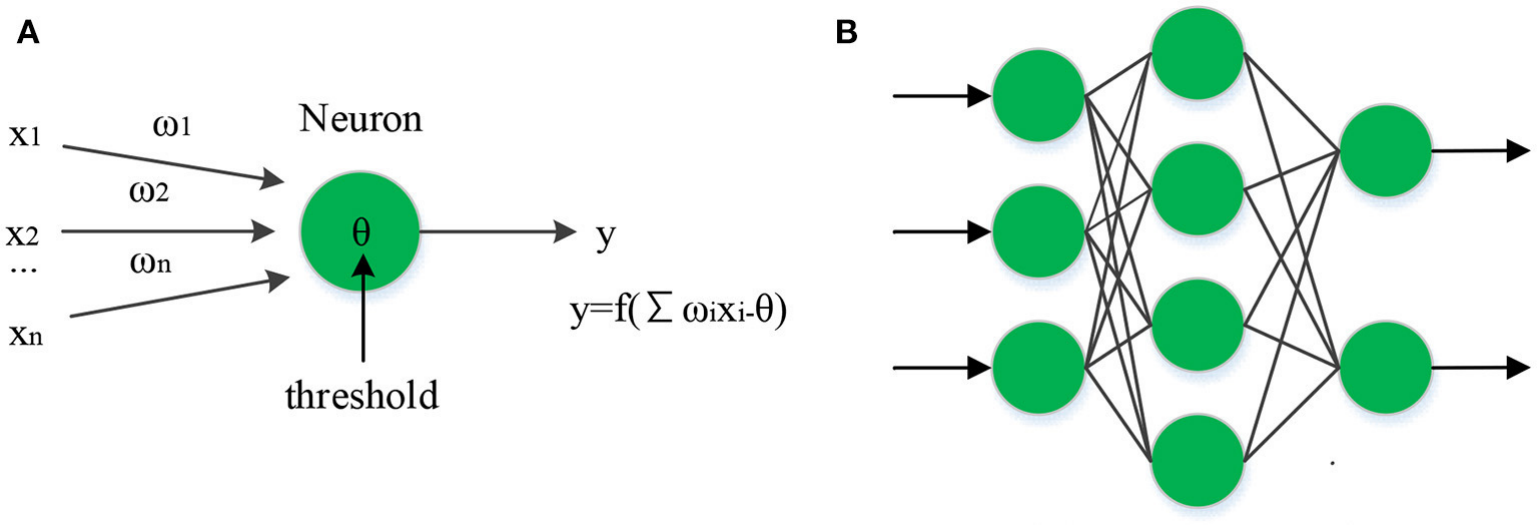
Model of backpropagation neuron and the two-layer structure diagram. **(A)** BP neuronal structure. **(B)** BP neural structure.

Mobile robots are widely used in unmodeled spaces and environments; thus, accurate trajectory tracking is the basis of practical applications (Alshakarchi and Al-Maliky, 2018; Singh and Thongam, 2018; Tu et al., 2019; Wang et al., 2019). The traditional control method depends too much on the dynamic model, resulting in low robustness. The intelligent neural networks have strong robustness and adaptability, presenting significant advantages in trajectory tracking and control of mobile robots. As the core of the feedforward network in artificial neural networks, BPNN is widely applied to solve problems such as function approximation and pattern recognition (Yi et al., 2019). The most commonly used transfer functions of backpropagation neurons are log function and tan function, and the output can be expressed as *y* = log *sig* (*Wp* + *b*). Generally, BPNN presents a multi-layer structure. The model of backpropagation neuron and the two-layer structure diagram are shown in Figure 3.

BPNN needs to adjust the weights according to each training sample, which requires a massive amount of training data in practical applications, resulting in reduced efficiency of weight adjustment and failure to meet the real-time requirements (Singh and Thongam, 2019). Therefore, the traditional BPNN is divided into several smaller sub-networks, which are trained separately to improve computational efficiency. While dividing the BPNN into *n* sub-networks, it is also necessary to divide the training samples into *n* groups of sub-samples. It is also necessary to compare the current training sample with the previous training sample to calculate the corresponding group's mean square error sum. Finally, according to the comparison result, whether to input the data into BPNN for operation is decided. The improved BPNN eliminates the need for repeated calculations, which significantly shortens the calculation time for large-scale neural networks and improves weight adjustment efficiency.

The improved BPNN is adopted to model the unknown parameters of the robot, and a dynamic controller that meets the real-time requirements of mobile robots is designed. First, the kinematic equation of the mobile robot is expressed as:

(11) $$\left\{ \begin{array}{l} \overset{\cdot }{x}=v\cos\\ \overset{\cdot }{y}=v\sin\\ \overset{\cdot }{\theta }=\omega \end{array}\right.$$

In Equation (11), (*x, y*) is the actual position of the mobile robot, θ is the azimuth angle, and both the linear velocity *v* and the angular velocity ω are control inputs in the kinematic model. Robot trajectory tracking is to track the target robot with pose $q_{r}={\left[x_{r},y_{r},\theta_{r}\right]}^{T}$ and velocity ${\overset{.}{q}}_{r}={\left[v_{r},\omega_{r}\right]}^{T}$. The tracking error of the mobile robot is expressed as:

(12) $$\begin{array}{l} e=\left[\begin{array}{c} e_{1}\\ e_{2}\\ e_{3} \end{array}\right]=T_{e}\left(q_{r}-q\right) \end{array}$$

The error change rate of the mobile robot can be expressed as:

(13) $$\begin{array}{l} \overset{.}{e}=\left[\begin{array}{c} {\overset{.}{e}}_{1}\\ {\overset{.}{e}}_{2}\\ {\overset{.}{e}}_{3} \end{array}\right]=\left[\begin{array}{c} \omega e_{2}-v+v_{r}\cos e_{3}\\ -\omega e_{2}+v_{r}\sin e_{3}\\ \omega_{r}-\omega \end{array}\right] \end{array}$$

The improved BPNN is applied to robot dynamics control. If a vector *P* (·) is a variable function, a static neural network is utilized for modeling, and the following equation will be obtained:

(14) $$\begin{array}{l} P\left(\cdot \right)=\left[{\left\{W_{P}\right\}}^{T}\cdot \left\{\xi_{P}\left(\cdot \right)\right\}\right]+E_{P}\left(\cdot \right) \end{array}$$

In Equation (14), ${\left\{W_{P}\right\}}^{T}$ and {ξ_*P*_ (·)} are OpenGL vectors, each element is a model error vector, and (·) represents a general vector or matrix.

### Robot Trajectory Tracking Based on the Fuzzy Algorithm Combining Neural Networks

In fuzzy systems, the design of fuzzy sets, membership functions, and fuzzy rules are based on empirical knowledge. This analysis method has a lot of subjectivity (Lu et al., 2018). Hence, the learning mechanism is introduced into the fuzzy system to modify and improve the membership function and fuzzy rules through continuous learning. The connection between the fuzzy system and the fuzzy neural network shows that the fuzzy neural network is essentially the realization of the fuzzy system. The difference between the two reveals that the fuzzy neural network has the characteristics of the neural network. Introducing the learning ability of the neural network into the fuzzy system and representing the fuzzy processing, fuzzy reasoning, and precise calculation of the fuzzy system through a distributed neural network is an important way to realize the self-organization and self-learning of the fuzzy system (Amador-Angulo et al., 2016). In a fuzzy neural network, the input and output nodes are used to fuzzify the input and output signals of the system (Caraveo et al., 2017; Lagunes et al., 2019). The hidden nodes of this neural network express the membership function and fuzzy rules, and the parallel processing capability of the neural network makes the inference ability of the fuzzy system greatly improved. The fuzzy neural network combines fuzzy system and neural network. A fuzzy neural network is essentially a conventional neural network that assigns fuzzy input signals and fuzzy weights. Its learning algorithm is usually a typical neural network's learning algorithm or its extension.

According to the kinematic model of the non-holonomic mobile robot, the current pose of the robot can be obtained as long as *u* = [*v ω*]^*T*^ is controlled. Assuming that the actual pose of the robot is *p* = [*x y θ*]^*T*^, the actual motion velocity is [*v ω*]^*T*^; the reference pose is $p_{r}={\left[x_{r}\;y_{r}\;\theta_{r}\right]}^{T}$, and the reference motion velocity is ${\left[v_{r}\;\omega_{r}\right]}^{T}$. Then the error vector between the actual pose and the reference pose is $p_{e}={\left[x_{e}\;y_{e}\;\theta_{e}\right]}^{T}$. Essentially, trajectory tracking of a non-holonomic mobile robot is to find a bounded input for any initial pose and velocity error and make:

(15) $$\begin{array}{l} \underset{t\to \infty }{\lim}||{\left[x_{e}\;y_{e}\;\theta_{e}\right]}^{T}||=0 \end{array}$$

The error vector between the actual pose and the reference pose can be expressed as:

(16) $$\begin{array}{l} p_{e}={\left[\begin{array}{c} x_{e}\\ y_{e}\\ \theta_{e} \end{array}\right]}^{T}=\left[\begin{array}{ccc} \cos\theta & \text{sin}\theta & 0\\ -\text{sin}\theta & \;\cos\theta & 0\\ 0 & 0 & 1 \end{array}\right]\cdot \left[\begin{array}{c} x_{r}-x\\ y_{r}-y\\ \theta_{r}-\theta \end{array}\right] \end{array}$$

Equation (16) is derived to obtain the differential equation of the mobile robot's tracking error, expressed as:

(17) $$\begin{array}{l} \left[\begin{array}{c} {\overset{\cdot }{x}}_{e}\\ {\overset{\cdot }{y}}_{e}\\ {\overset{\cdot }{\theta }}_{e} \end{array}\right]=\left[\begin{array}{c} v_{r}\cos\theta_{e}-v+y_{e}\omega \\ v_{r}\text{sin}\theta_{e}-x_{e}\omega \\ \omega_{r}-\omega \; \end{array}\right] \end{array}$$

**Figure 4:**
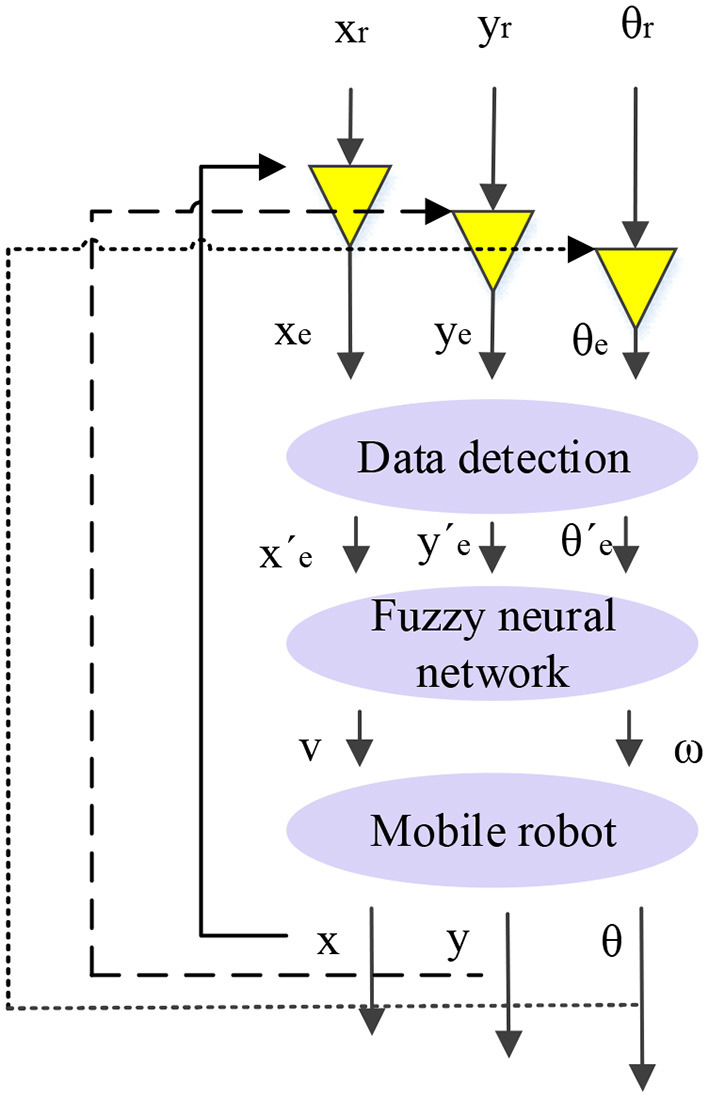
A fuzzy-BPNN trajectory tracking controller for mobile robots.

According to the above principles and derivations, a fuzzy-BPNN trajectory tracking controller for mobile robots is designed, as shown in Figure 4. It can control the linear velocity and angular velocity of mobile robots.

The actual trajectory tracking process will be disturbed by obstacles and other external environmental factors. Hence, the pose error will change significantly (Boujelben et al., 2017; Lu et al., 2017; Bencherif and Chouireb, 2019). Fuzzy logic can imitate the thinking way of the human brain and process systems with unknown models. Therefore, fuzzy logic can determine the location of obstacles while mobile robots are moving. Its core is to process the pose error data to avoid problems such as an increased number of fuzzy neural network's rules and the repeated change of weights. A data detection step is added, which uses the averaged error changes of the previous pose as a reference and compares it with the new pose error for decision-making. The data detection step can eliminate the error data with apparent mutations in the input. The fuzzy neural network's parameters do not need to be adjusted significantly. Therefore, the stability of the system and the overall calculation efficiency can be improved.

### IoT Multi-Sensor Data Fusion and Image Enhancement

A primary function of IoT technology is information perception. The object is connected to the network for information exchange through the installed sensors, electronic tags, and other sensing devices, realizing intelligent identification, positioning, and supervision operations. In the process of information collection, the mobile robots' sensors summarize multiple sensors' data through data fusion, thereby reducing the transmission of redundant information and improving the stability and accuracy of the system (Jing et al., 2017).

**Figure 5:**
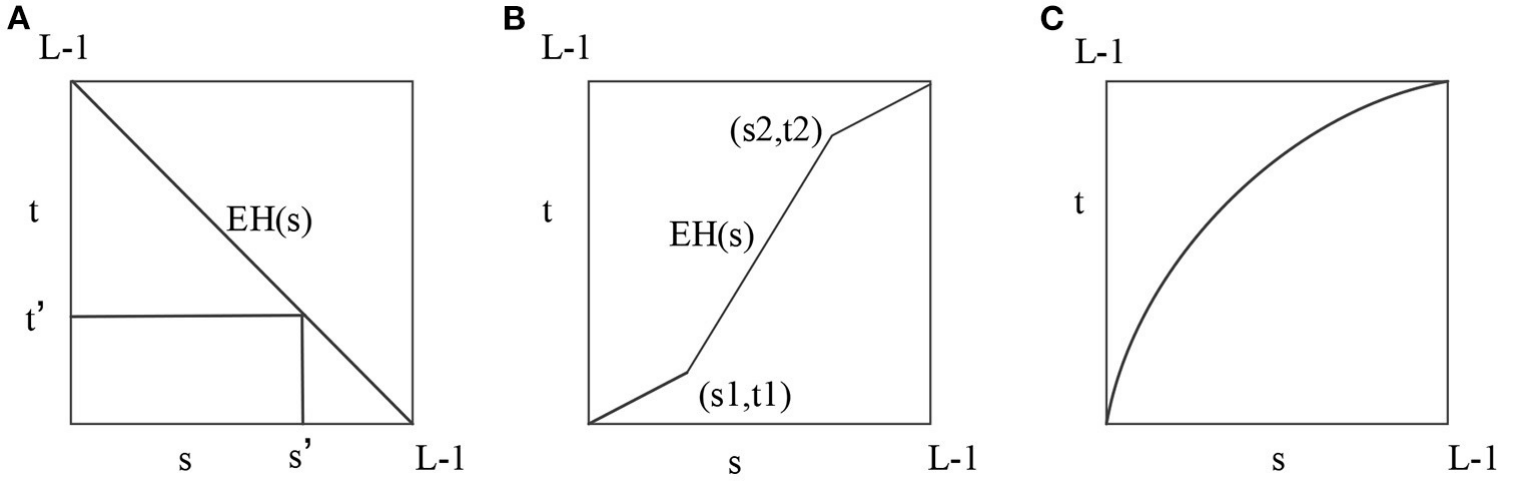
Principle of each image enhancement step. **(A)** Inversion of gray scale. **(B)** Enhance contrast. **(C)** Dynamic image compression.

IoT intelligent image enhancement establishes an image enhancement model through a wavelet conversion scale, which can adaptively adjust the window of different digital image frequencies to intelligently and adaptively enhance the IoT images. To improve the accuracy of environmental information collection, a mobile robot often uses an adaptive fusion algorithm to process the environmental information collected by its system (Yamashita et al., 2017; Ravi and Krishnan, 2018; Fan et al., 2019). Since the background of the original image collected by the infrared thermal imager and the monitored target robot are not notable, the details of the target image are difficult to identify, and the image feature cannot be clearly extracted. Hence, the image enhancement method is adopted to process the target image. The grayscale transformation method is adopted to enhance the image's contrast and improve its visual effects. Standard methods include (1) direct grayscale transformation; (2) transformation with the help of histogram; (3) transformation with a series of operations between images (such as addition and subtraction) (Chen et al., 2020). The direct grayscale transformation is the most commonly used and most convenient method. First, in the process of negating the gray, the image needs to be negated; that is, to reverse the gray value of the original image. Second, the image's contrast enhances the contrast of each part of the original image. Sometimes the dynamic range of the original image can exceed the allowable range of display devices. Therefore, if the original image is used directly, some details will be lost (Long et al., 2018; Singh et al., 2018). The solution is to compress the original image in grayscale. The principle of grayscale negation, contrast enhancement, and dynamic image compression is shown in Figure 5.

Moreover, image noise processing is vital in image enhancement, including impulse noise and Gaussian noise. Usually, image denoising separates the image into two kinds of noise; then, the median filter algorithm and the mean filter algorithm are used to eliminate these noises. The median filter algorithm arranges the to-be-processed pixels' gray values in the neighborhood from large to small. Then it selects the median value to replace the to-be-processed pixel value in the template center. The mean filtering algorithm removes the sudden change by calculating the mean value of a central point and several surrounding points, thereby removing the noises.

### Simulations Experiments

MATLAB is chosen as the simulation software to analyze the performances of the improved BPNN and the fuzzy-BPNN in robot trajectory tracking and control. MATLAB is one of the excellent science and technology application software. It has powerful calculation and visualization functions but is simple and easy to operate. In particular, the accompanying toolbox that supports more than 30 different fields has made it the basic tool and preferred platform for computer-aided design and analysis in various fields.

The performance differences between the two algorithms are compared. The reference linear velocity of the target robot is set to *v*_*r*_ = 3 m/s, the reference angular velocity is set to ω_*r*_ = 2 rad/s, the initial pose is set to [*x*_*r*_ *y*_*r*_ θ_*r*_] = (−5, 0.25, 0), and the initial pose error is set to ${\left[e_{1}\;e_{2}\;e_{3}\right]}^{T}={\left[2.6\;2.4\;\pi /2\right]}^{T}$. Ten groups of samples are taken for training. Each group of samples is divided into five groups of sub-samples equally. The mean square errors of the corresponding sub-samples of each group of samples (Table 1) are summed. The errors eventually converge to 0, the convergence speed is fast, and satisfactory results can be achieved.

**Table 1:** The sum of the mean square errors of the sub-samples of each training sample.

**Training samples**	**(1)**	**(2)**	**(3)**	**(4)**	**(5)**
Group 1	0	0	0	0	0
Group 2	3.4	53.5	6.6	12.3	78.9
Group 3	11.3	5.6	5.3	4.3	53.5
Group 4	30.4	11.2	54.5	45.7	6.6
Group 5	6.9	22.3	34.1	23.6	5.9
Group 6	12.5	91.3	1.8	66.5	45.6
Group 7	34.5	11.7	7.2	4.8	12.3
Group 8	88.9	23.5	4.7	67.8	3.4
Group 9	2.2	23.4	44.6	32.4	11.1
Group 10	77.4	32.5	38.8	44.6	65.4

## Results and Discussion

### Trajectory Tracking Performance of Fuzzy-BPNN

**Figure 6:**
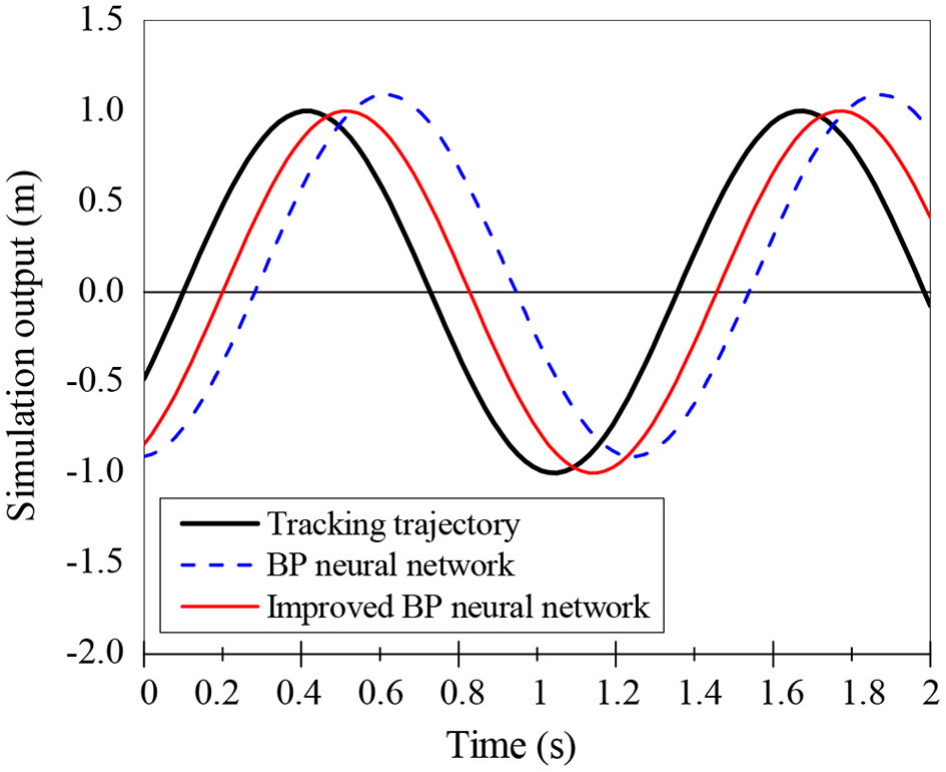
Trajectory tracking effect of the improved BPNN algorithm.

**Figure 7:**
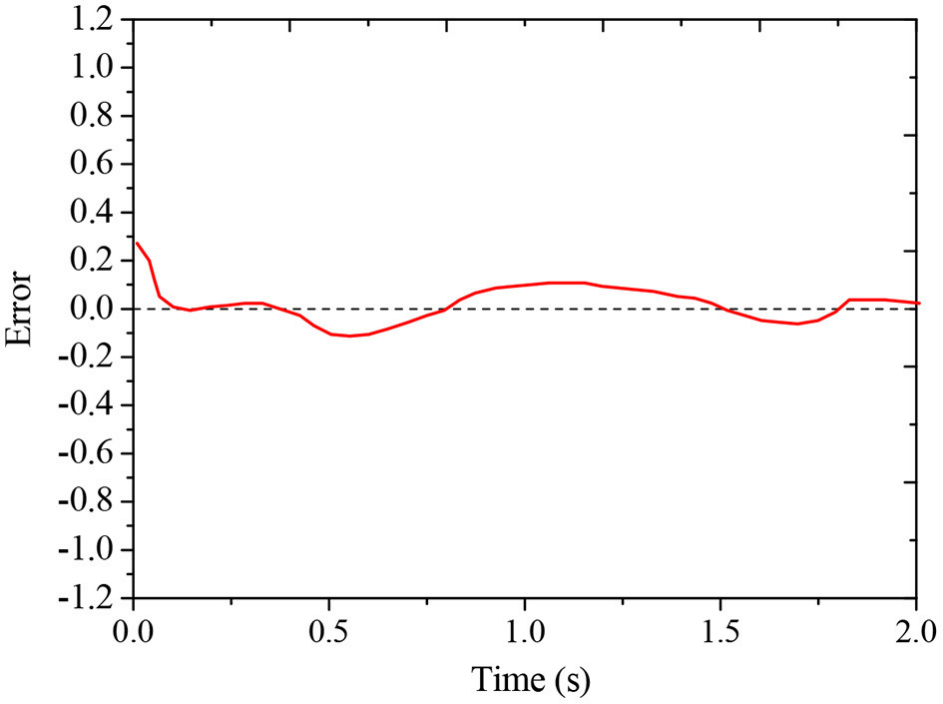
Trajectory tracking error curve of the improved BPNN algorithm.

The effects of traditional BPNN and improved BPNN in mobile robots' trajectory tracking are compared, and the results are shown in Figures 6 and 7. The improved BPNN algorithm has a slight improvement in tracking accuracy than the traditional BPNN; however, the difference between the two is not notable. On the contrary, the improved BPNN algorithm has a significant improvement in training speed, showing its performance advantages in processing huge samples in reality.

**Figure 8:**
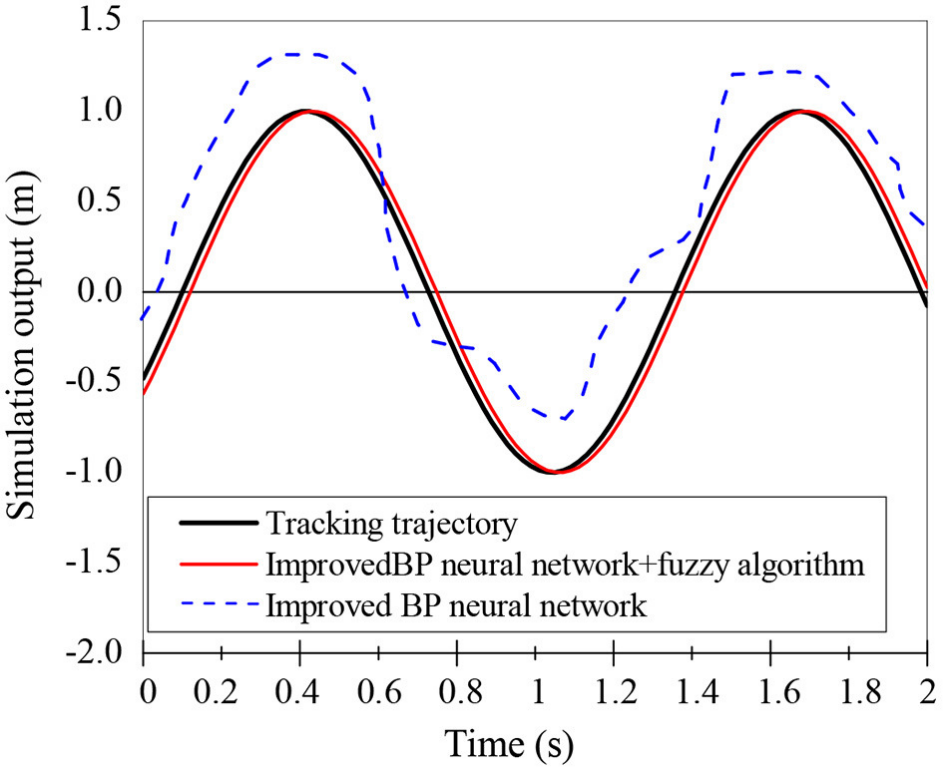
Trajectory tracking effect of fuzzy-BPNN.

The fuzzy-BPNN algorithm is applied to track the mobile robot's trajectories, whose effects are compared with the improved BPNN. The simulation results are shown in Figure 8. The fuzzy-BPNN controller has significantly improved tracking speed and tracking accuracy compared with the improved BPNN, proving its effectiveness.

### Non-holonomic Mobile Robot's Trajectory Tracking and Control Results

**Figure 9:**
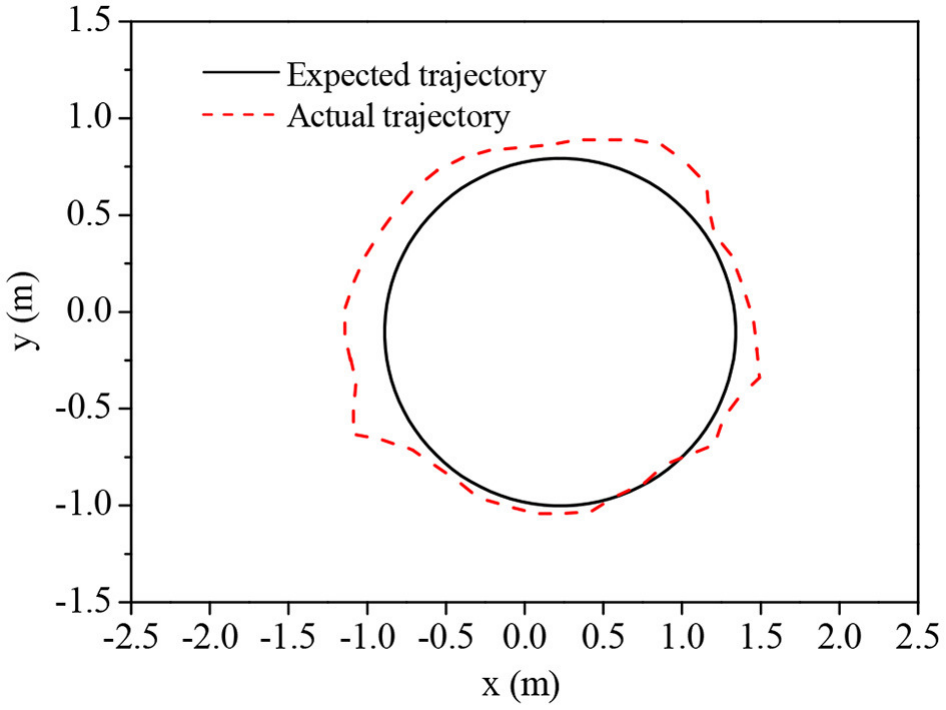
Circular trajectory's tracking effect.

**Figure 10:**
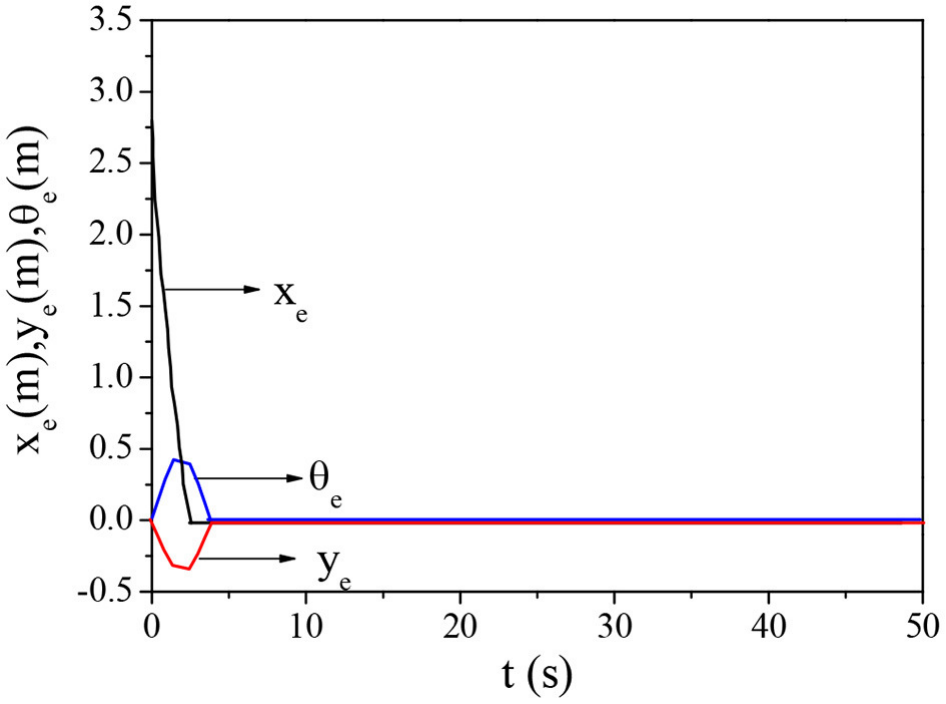
Changes in pose error in circular trajectory tracking.

**Figure 11:**
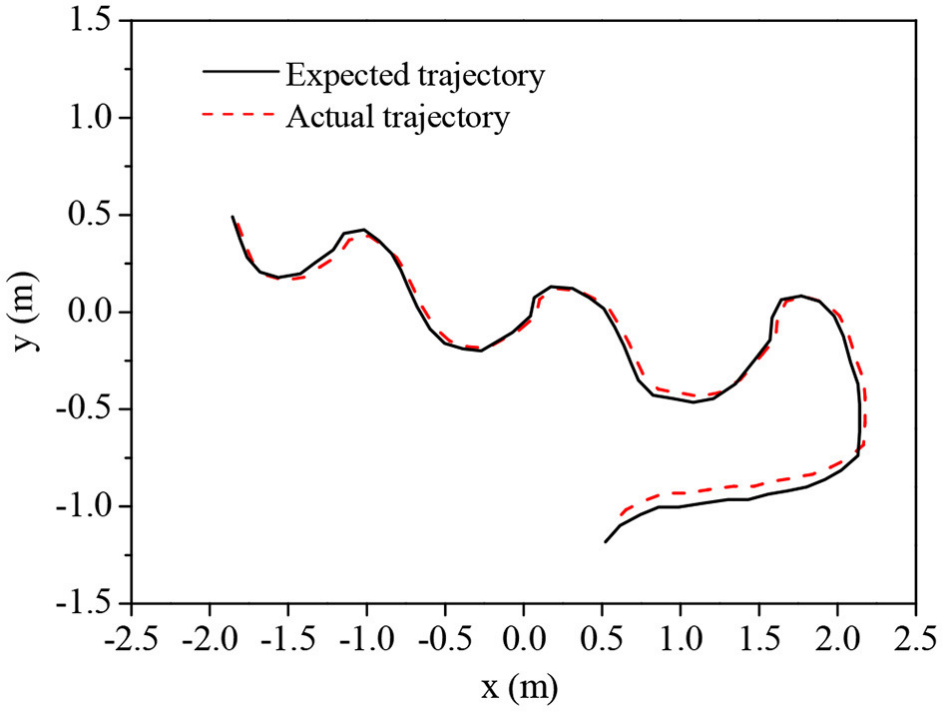
Curve trajectory's tracking effect.

**Figure 12:**
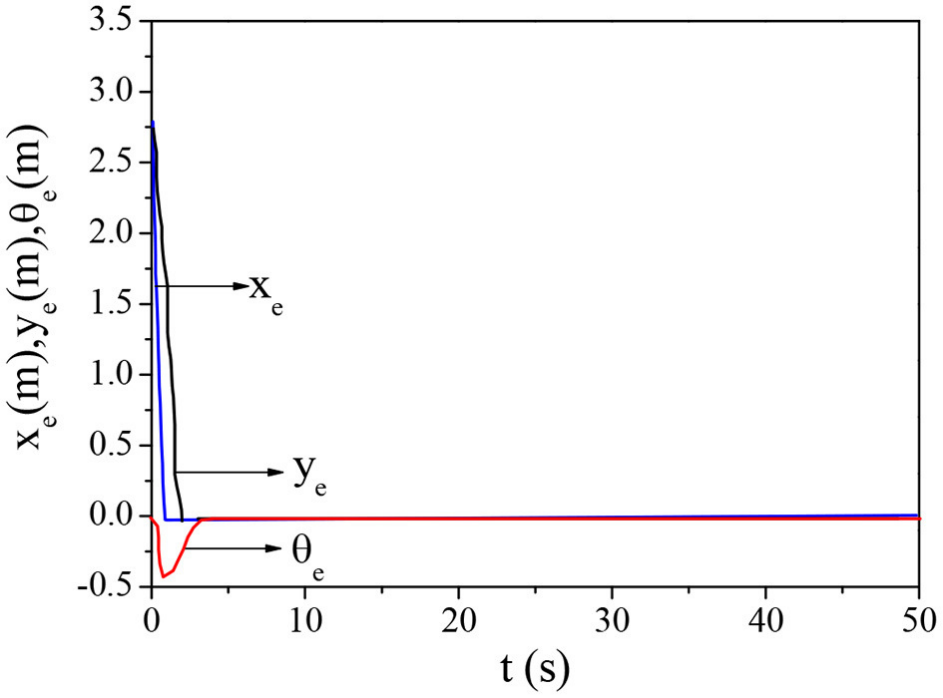
Changes in pose error in curve trajectory tracking.

The designed fuzzy-BPNN is integrated with the sliding mode trajectory tracking controller to solve the trajectory tracking and control problems of non-holonomic mobile robots. The tracking and control of typical circular and curved trajectories are simulated. Figures 9 and 10 show the tracking effects and pose error changes in circular trajectory tracking, and Figures 11 and 12 demonstrate the tracking effects and pose error changes in curve trajectory tracking. The designed algorithm integrating the sliding mode trajectory tracking controller shows good trajectory tracking and control effects for these two different trajectories.

## Conclusion

As an essential branch of robotics, mobile robots can continuously obtain the surrounding environment's status through sensors to make decisions and complete highly intelligent tasks. Robot intelligence is embodied in planning and executing an optimal path for mobile robots. Its core is trajectory tracking and control. Here, the mathematical kinematics models of non-holonomic mobile robots are analyzed first. Then the advantages of BPNN being widely used in pattern recognition and other problems are discussed. The traditional BPNN is improved, considering loads of data need to be trained in practical applications. The computational efficiency is improved by dividing BPNN into several smaller sub-networks for separate training. A fuzzy-BPNN tracking controller for mobile robot trajectories is designed to control the robot's linear velocity and angular velocity, in an effort to improve the accuracy of trajectory tracking. In addition, adaptive fusion algorithms and image enhancement techniques are used to process the environmental information collected by mobile robots to improve the accuracy of the environmental information collection.

A simulation experiment is run in the MATLAB environment to analyze the performances of the improved BPNN and the fuzzy-BPNN in robot trajectory tracking and control. Compared with traditional BPNN, the improved BPNN algorithm has a significant improvement in training speed, which has better application value for the large sample problems in reality. The fuzzy-BPNN controller has notably improved the tracking speed and accuracy compared with the improved BPNN algorithm. The designed fuzzy-BPNN algorithm is integrated with the sliding mode trajectory tracking controller, whose performances in tracking and controlling circular and curve trajectories are simulated. The designed fuzzy BPNN algorithm is integrated with the sliding mode trajectory tracking controller. The circular and curved trajectories are simulated, and both show good trajectory tracking and control effects. However, the external environment is complicated and changeable; maintaining the stability of the mobile robot controller will be the principal direction of the following research.

## Data Availability Statement

The raw data supporting the conclusions of this article will be made available by the authors, without undue reservation.

## Author Contributions

All authors listed have made a substantial, direct and intellectual contribution to the work, and approved it for publication.

## Conflict of Interest

The authors declare that the research was conducted in the absence of any commercial or financial relationships that could be construed as a potential conflict of interest.
